# Strategies and utility of imputed SNP genotypes for genomic analysis in dairy cattle

**DOI:** 10.1186/1471-2164-13-538

**Published:** 2012-10-08

**Authors:** Mehar S Khatkar, Gerhard Moser, Ben J Hayes, Herman W Raadsma

**Affiliations:** 1 Reprogen - Animal Bioscience, Faculty of Veterinary Science, University of Sydney, 425 Werombi Road, Camden, NSW, 2570, Australia; 2 Department of Primary Industries, Biosciences Research Division, Bundoora, Victoria, 3083, Australia; 3 Dairy Futures Cooperative Research Centre (CRC), Bundoora, Victoria, Australia

**Keywords:** Imputation, 800K, High-density SNP, Dairy cattle, Genomic selection

## Abstract

**Background:**

We investigated strategies and factors affecting accuracy of imputing genotypes from lower-density SNP panels (Illumina 3K, 7K, Affymetrix 15K and 25K, and evenly spaced subsets) up to one medium (Illumina 50K) and one high-density (Illumina 800K) SNP panel. We also evaluated the utility of imputed genotypes on the accuracy of genomic selection using Australian Holstein-Friesian cattle data from 2727 and 845 animals genotyped with 50K and 800K SNP chip, respectively. Animals were divided into reference and test sets (genotyped with higher and lower density SNP panels, respectively) for evaluating the accuracies of imputation. For the accuracy of genomic selection, a comparison of direct genetic values (DGV) was made by dividing the data into training and validation sets under a range of imputation scenarios.

**Results:**

Of the three methods compared for imputation, IMPUTE2 outperformed Beagle and fastPhase for almost all scenarios. Higher SNP densities in the test animals, larger reference sets and higher relatedness between test and reference animals increased the accuracy of imputation. 50K specific genotypes were imputed with moderate allelic error rates from 15K (2.85%) and 25K (2.75%) genotypes. Using IMPUTE2, SNP genotypes up to 800K were imputed with low allelic error rate (0.79% genome-wide) from 50K genotypes, and with moderate error rate from 3K (4.78%) and 7K (2.00%) genotypes. The error rate of imputing up to 800K from 3K or 7K was further reduced when an additional middle tier of 50K genotypes was incorporated in a 3-tiered framework. Accuracies of DGV for five production traits using imputed 50K genotypes were close to those obtained with the actual 50K genotypes and higher compared to using 3K or 7K genotypes. The loss in accuracy of DGV was small when most of the training animals also had imputed (50K) genotypes. Additional gains in DGV accuracies were small when SNP densities increased from 50K to imputed 800K.

**Conclusion:**

Population-based genotype imputation can be used to predict and combine genotypes from different low, medium and high-density SNP chips with a high level of accuracy. Imputing genotypes from low-density SNP panels to at least 50K SNP density increases the accuracy of genomic selection.

## Background

Innovations in genomic technologies provide new tools for enhancing productivity and wellbeing of domestic animals. Genomic selection, where genetic merit is predicted from genome-wide single nucleotide polymorphism (SNP) genotypes [1,2], is used in the dairy industries in a number of countries [3,4]. The rapid uptake of this technology has been driven by both the availability of commercial high-density SNP chips, and increased genetic gain over traditional progeny testing largely as a consequence of reduced generation interval and increased accuracy of selection at a younger age [5-7].

A number of SNP chips from Illumina ( http://www.illumina.com) and Affymetrix ( http://www.affymetrix.com) are available for cattle. These include 3K [8], 7K [9], 15K [10], 25K [11], 50K [12] and more recently 800K from Illumina, and 650K and 3 million SNP panels from Affymetrix. In addition next generation sequencing technologies for low-cost sequencing of whole genomes are now available [13]. Use of genotypic data from high-density SNPs potentially can increase accuracy of genomic selection but also the total cost of genotyping/sequencing. As new higher density chips are developed, re-genotyping previously genotyped samples or new samples with new chips or whole genome sequencing is expensive. For some applications, such as selection of heifers to be retained in the dairy herd or selection in beef production systems, low-density SNP panels e.g. 3-7K may be the only cost effective option (e.g. [14]). If low-cost genotyping could be useful, very large numbers of animals can be genotyped on a routine basis.

Accuracy of genomic predictions based on different subsets of low-density SNP panels up to 50K have been compared in a number of studies [15-18]. A common finding is that accuracy of genomic prediction for young animals increased as the number of markers increased from a few hundred up to all SNPs from 50K SNP chip. There are several possible strategies how to select loci for low-density panels [17]. However, instead of using lower density SNP in genomic prediction, a promising approach is to genotype a small proportion of the population with a high-density SNP panel and then employ genotype imputation methods for predicting high-density genotypes for the rest of the population genotyped with a lower density SNP panel (e.g. [8,9]). Genotypic imputation is defined as the prediction of genotypes at the SNP locations in a sample of individuals for which assays are not directly available. These *in silico* genotypes obtained by imputation, *albeit* with some uncertainty, can then be used in genome-wide association and genomic selection analyses (e.g. [19,20]). Such strategies are likely to result in more accurate predictions of genomic breeding values [21], improved ability to resolve or fine-map QTL or QTN, and integration and meta-analysis across large datasets with heterogeneous SNP information [22].

A number of imputation software programs (fastPHASE [23], MACH [24], IMPUTE [25], Beagle [19], PLINK [26], DualPhase [27]) have been used to infer missing or untyped genotypes based on known information derived from flanking markers. A number of studies on imputing genotypes have been published in dairy cattle [21,28-33] using 50K data and more recently high-density SNP panels [34-36] reporting accuracies of imputation from lower SNP panels to 50K and up to high-density SNP panels examining different methods of imputation, often using limited number of scenarios and strategies of using test and reference panels. The direct comparisons across such studies are thus often difficult. Various factors affecting the accuracy of imputation require further systematic investigation. The accuracy of imputation can be improved by increasing the size of the reference population [37]. For some resource population the animals genotyped with different SNP panels are available. Such genotype resources can be better utilised by imputing in a tiered framework, utilising multiple reference panels, which might result in improved accuracy of imputation in the study samples [38].

The objectives of this study were to evaluate the accuracies of imputation using three different population based methods of imputation, different size of reference and test panels, different imputation strategies, different SNP array platforms, effect of relationship between reference and test animals and finally examine the effect of using imputed genotypes on the accuracy of genomic selection.

## Methods

### Data

In total four datasets genotyped with four different SNP chips (Table 1) were used. The largest dataset consisted of 2,727 (2,205 bulls and 522 cows) Australian Holstein-Friesian cattle [17] genotyped with Illumina BovineSNP50 BeadChip [12]. A second more recent dataset consisted of 845 Australian Holstein-Friesian heifers genotyped with Illumina 800K BovineHD beadChip (Illumina Inc., San Diego, CA). After applying quality control (minor allele frequencies (MAF) >0.01, call rate>0.9, Hardy Weinberg Equilibrium (HWE) P>0.0001) a total of 42,136 and 610,879 autosomal SNPs from the 50K and the 800K chips, respectively, were used in the present study (Table 1). In addition any genotype showing Mendelian inconsistencies was set to missing.

**Table 1:** Description of different SNP chips and SNP subset panels

**Label used for SNP panel in this study**	**SNP chip**	**Number of SNPs on chip**	**Filtered SNPs used in this study**	**Remarks**
15K	15K (ParAllele/Affymatrix)	15,036	205 SNPs from BTA20	
25K	25K (Affymatrix)	25,068	328 SNPs from BTA20	
50K	Illumina BovineSNP50 BeadChip	54,001	42,136	
3K	Illumina BovineSNP50 BeadChip	3,000	3,000	Evenly spaced Subset of 50K
5K	Illumina BovineSNP50 BeadChip	5,000	5,000	Evenly spaced Subset of 50K
10K	Illumina BovineSNP50 BeadChip	10,000	10,000	Evenly spaced Subset of 50K
20K	Illumina BovineSNP50 BeadChip	20,000	20,000	Evenly spaced Subset of 50K
35K	Illumina BovineSNP50 BeadChip	35,000	35,000	Evenly spaced Subset of 50K
BovineLD 7K	Illumina BovineLD BeadChip	6,909	6,662	
Bovine3K	Illumina Bovine3K BeadChip	2,900	2,500	
800K	Illumina 800K BovineHD beadChip	786,799	610,879	
800K-imputed	Illumina 800K BovineHD beadChip	786,799	610,879	Imputed best guess genotypes
800K-dosage	Illumina 800K BovineHD beadChip	786,799	610,879	Imputed dosage for B-allele
49K	Illumina BovineSNP50 BeadChip	54,001	49,394	Common SNP between 800K and 50K chip

Of the 2,205 bulls with 50K genotypic information, 1,419 were previously genotyped for 15K [10], and 431 for 25K ( [11], http://www.affymetrix.com). These datasets were used to test the accuracies of imputing SNP genotypes between different chips. The animals in all these datasets are related in a complex pedigree structure. The distributions of relatedness in the form of boxplots of pedigree kinship among animals in different datasets are given in Additional file 1.

### Imputation Scenarios

Animals were divided into reference and test sets for evaluating the accuracies of imputation. The animals included in the reference set have genotypes derived from the high-density SNP panel and the animals in the test set have genotypes from the lower density SNP panel. The lower density SNP panels of the test sets were created by using a subset of the genotyped SNPs. The rest of the genotypes of the test sets were masked and used to compute the accuracy of imputation. A number of imputation scenarios were generated by combining different reference and test sets and SNP densities. The animals (2,727) genotyped with 50K were divided into 8 different combinations of reference and test sets as presented in Table 2. Reference animals in reference-test-ID 1–4 are a random sample of older bulls born before 2001. The 27 bulls for reference-test-ID 5 are key ancestors of the Australia Holstein-Friesian population. In reference-test-ID 8, younger bulls born between 2001 and 2004 are in the test set and all older bulls born before 2001 in the reference set.

**Table 2:** Composition of reference and test sets for evaluating imputation accuracy up to 50K

**Reference-test-ID**	**Data**	**Reference set**			**Test set**			**Total (animals)**
		**n**	**%**	**Description**	**n**	**%**	**Description**	
1	50K	1363	50	bulls	1364	50	bulls+cows	2727
2	50K	681	25	bulls	2046	75	bulls+cows	2727
3	50K	272	10	bulls	2455	90	bulls+cows	2727
4	50K	136	5	bulls	2591	95	bulls+cows	2727
5	50K	27	1	key bulls	2700	99	bulls+cows	2727
6	50K	2205	81	all bulls	522	19	all cows	2727
7	50K	522	19	all cows	2205	81	all bulls	2727
8	50K	1753	80	training set bulls	452	20	test set young bulls	2205

The total number of animals (2,727) consisted of 2,205 bulls and 522 cows.

To examine the effect of pedigree relatedness between test and reference animals on the accuracy of imputation, the test animals with sire and without sires in the reference set were compared. In addition the highest value of pedigree kinship for each test animal with reference animals was computed. The test animals were classified into four interval categories with respect to their highest pedigree kinship viz. 0.0-0.01, 0.01-0.1, 0.1-0.2 and 0.2-0.4. The accuracy of imputation of the test animals in these four categories was compared using IMPUTE2.

**Figure 1:**
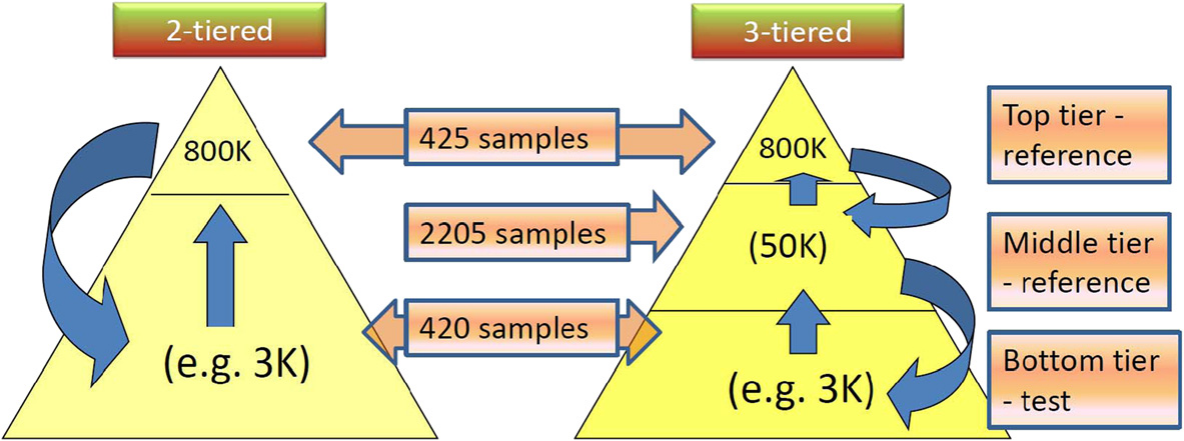
**Comparison of 2-tiered and 3-tiered imputation framework.** The 2-tiered framework is composed of top tier (reference panel) and lower tier (test panel). Three separate test panels (bottom tier) using three SNP densities, viz. Bovine3K, BovineLD 7K and 50K, were analysed. In 3-tiered framework an additional panel of 2205 samples with 50K genotypes is included as middle tier.

For the 800K dataset, the 845 heifers were randomly divided in two subsets of approximately equal size *i.e.* 425 in the reference and 420 in the test set. This framework of imputation is referred here as a ‘2-tiered’ framework. This was extended to a ‘3-tiered’ framework by including an additional panel of 2,205 bulls with 50K SNP genotypes as a middle tier (Figure 1). An additional scenario using fewer animals in the top-tier was generated by randomly selecting 45 out of 425 reference heifers. The imputation for the tiered framework was performed with IMPUTE2 using the two reference panels in the same run.

### Generating low-density SNP panels

To mimic various low-density SNP panels, different subset of 50K SNPs were selected for the test sets. The SNP densities equivalent to 3000, 5000, 10000, 20000 and 35000 evenly spaced autosomal SNPs were generated by iterative thinning the SNPs based on spacing and MAF of SNPs (Table 1). In each iteration, a SNP pair with the smallest interval was identified and the SNP with lower MAF was removed from the pair. A total of 1,324 SNPs on chromosome 20 from the 50K panel were used for the initial analyses to compare the imputation programs for different scenarios. The best method of imputation identified was then used for analysing all the autosomal SNPs from the Illumina Bovine3K and Illumina BovineLD 7K BeadChip (Illumina Inc., San Diego, CA) for assessing the comparative utility of imputed genotypes from these commercial panels up to 50K for genomic prediction.

Most of the SNPs on the 50K chip are present on the 800K chip. For the scenarios using the 800K panel the lower density SNP panels for the test set consisted of common SNPs between 800K and 50K as well as between 800K and Illumina Bovine3K and Illumina BovineLD 7K, respectively (Table 1).

### Imputation methods

Population based imputation methods rely on linkage disequilibrium relationship between SNPs, and essentially consist of two steps viz. inference of haplotypes and imputing untyped genotypes in the test set using information from the best fit haplotypes derived from the reference panel. We compared three commonly used population-based programs for imputing missing genotypes which don’t rely on pedigree information viz. IMPUTE2, fastPhase and Beagle.

We used IMPUTE2 version 2.1.2 in this study which implements a Hidden Markov Model (HMM). The details of the algorithm are given in [25].The algorithm involves estimating haplotypes using all the SNP in reference set and then imputing the alleles at untyped SNPs in the test set based on the best fit haplotypes estimated from the reference. IMPUTE2 requires to specify the effective population size as an input parameter. This was set to 100 which is within the range of the effective population size reported for Holstein-Friesian dairy cattle [39,40].

We used fastPHASE version 1.2.3 [23]. fastPhase uses a haplotype clustering algorithm which is based on the observation that haplotypes in a population tend to cluster into groups of closely related or similar haplotypes over a short region. fastPhase requires the number of clusters K as input and was set to 20 in this study.

Beagle version 3.3 is also based on a local haplotype-clustering model (as detailed in [19], [37]), similar to fastPHASE, but allows for a variable number of clusters across a region. Beagle uses a localized haplotype cluster-model to cluster haplotypes at each marker and then defines a HMM to find the most likely haplotype pairs based on the individual’s known genotypes. The most likely genotype at untyped loci is generated from defined haplotype pairs. We used the option where reference and test panel are defined separately. Imputation was performed for each chromosome separately for all the three methods. Except the above mentioned parameters, programs were run with default parameters.

### Accuracy of imputation

All the three imputation methods provide the probability of the three possible genotypes at each missing genotype. We used the most likely genotype as the predicted genotype. For incorrectly imputed genotypes it is possible to impute one or both alleles incorrectly. To distinguish between these two cases, we computed the accuracy of imputing alleles as the percentage of correctly predicted alleles, and the allelic error rate of imputation as the percentage of incorrectly predicted alleles *i.e.* mean allelic error rate (%) = number of incorrectly predicted alleles / total number of alleles imputed in the test set × 100. In general allelic error rates are just slightly more than half of genotypic error rates. Accuracy of imputation was also computed as the percentage of correctly predicted genotypes for the masked genotypes.

### 800K imputed dataset for genomic prediction

The data on 2,205 bulls genotyped with 50K were imputed, with IMPUTE2, up to 800K using 845 heifers genotyped with 800K as reference and using most likely genotype as the predicted genotype (‘800K-imputed’, Table 1). In addition the dosage/copies of the B allele for each genotype was computed as *p*_*AB*_+2×*p*_*BB*_, where *p*_*AB*_ and *p*_*BB*_ are imputed probabilities of AB and BB genotypes, respectively. This measure takes into account the uncertainty of imputation and is an appropriate measure when using an additive model in genomic prediction and genome-wide association studies. These two datasets of 2,205 bulls with imputed genotypes (‘800K-imputed’) and imputed dosage (‘800K-dosage’) for 610,879 autosomal SNPs were used to compute genomic prediction.

### Accuracy of genomic prediction

Accuracy of direct genetic values (DGV) using imputed and actual genotypes was investigated by dividing the data on 2,205 bulls in a training set of 1,753 bulls born between 1955 and 2000 and a validation/test set of 452 young bulls born between 2001 and 2004. SNP effects were obtained from the solution of the following mixed model equations [41,16]

(1) $$\left[\begin{array}{c} 1'R^{-1}1\\ X'R^{-1}1 \end{array}\;\begin{array}{c} 1'R^{-1}X\\ X'R^{-1}X+\lambda I \end{array}\right]\left[\begin{array}{c} \hat{\mu }\\ \hat{g} \end{array}\right]=\left[\begin{array}{c} 1'R^{-1}y\\ X'R^{-1}y \end{array}\right]$$

where **y** is a vector of twice the daughter trait deviations (DTD) of bulls, 1 is a column vector of ones of size *N*_*Anim*_ , $\hat{\mu }$ is the general mean, *ĝ* is a vector of the estimated SNP effect, X is an *N*_*Anim*_ × *N*_SNP_ matrix of SNP genotypes coded as 0 (homozygote), 1 (heterozygote), or 2 (other homozygote), or SNP allele dosage. I is an identity matrix of size *N*_SNP_ × *N*_SNP_ , *λ* is a shrinkage parameter derived by cross-validation. R is a diagonal matrix with elements *R*_*ii*_ = (1/*rel*_*i*_)-1, where *rel*_*i*_ is the reliability of the DTD of ith bull. DGV were calculated as $\overset{⌢}{m}=\hat{\mu }+X\hat{g}$.

Five traits were analysed viz. milk yield, fat yield, protein yield, survival and daughter fertility which reflect a range of heritabilities (*i.e.* 0.25, 0.25, 0.25, 0.04 and 0.04, respectively). Phenotype information was provided by the Australian Dairy Herd Improvement Scheme (ADHIS, http://www.adhis.com.au). The phenotypes used were daughter trait deviations (DTD) for the bulls. The accuracy of the DGV prediction using subsets of SNP genotypes, and imputed SNP genotypes were compared to the DGV prediction obtained with the all 50K SNP genotypes. The accuracy of DGV prediction was computed as Pearson’s correlation coefficient between DGV and DTD of the young bulls in the test data.

## Results

### Imputation up to 50K

#### Comparison of imputation methods

**Figure 2:**
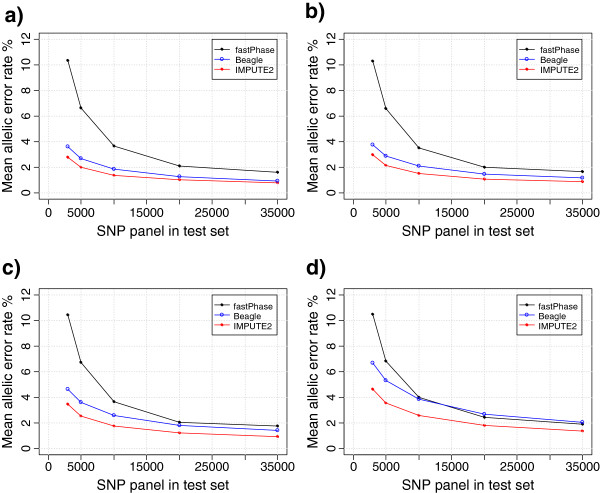
**Mean allelic error rate (%) of three imputation methods using different proportion of animals in reference and test sets for varying SNP density (3K-35K evenly spaced) in the test set.** The results shown are for chromosome 20.

The allelic error rates of imputing genotypes on BTA20 by the three imputation methods across different scenarios using evenly spaced SNP subsets in the test sets and different proportion of animals in the reference sets are presented in Figure 2. Detailed results on all the 42 scenarios are given in Additional file 2. In general IMPUTE2 has the lowest mean allelic error compared to Beagle and fastPhase, however, the difference between methods varies over different scenarios (Figure 2). The difference in error rate of IMPUTE2 and Beagle decreases with increasing size of the reference set and increasing SNP density in the test set (Figure 2). fastphase outperformed the other two methods in only one scenario where a higher SNP density (35K) was used in the test set and very few animals (27) were used as reference *i.e.* scenario 29 (Additional file 2). The accuracies of imputation of all the three haplotype based methods are much higher compared to imputation based on a simple sampling strategy using the allele frequencies of SNP in the reference set. The mean allelic error rates obtained from such sampling strategies are in the range of 22.5 to 26.8% for the different scenarios (Additional file 2).

#### Effect of SNP density

The accuracy of imputation increases with the number of SNPs in the test set (Figure 2, Additional file 2) for all the scenarios and the methods examined here. The mean allelic error rate decreases from 2.80% for the evenly spaced 3K SNP panel to 0.76% for the 35K panel in the scenario where 50% animals are in the reference set (Figure 2a). The mean allelic error rate of imputation is lower for the evenly spaced 3K SNP panel (2.80%) compared to the Bovine3K panel (3.34%). There is a large reduction in the mean allelic error rate of imputation when using the 5K evenly spaced SNP panel (1.97%) in the test set (Additional file 2). Further reductions in error rate of imputation by increasing SNP density in the test set to 10K (1.36%), 20K (1.00%) and 35K (0.76%) are relatively smaller (Figure 2a).

#### Effect of size of reference panel

The mean allelic error rate increases as the number of animals in the reference set decreases (Figure 2, Additional file 2). The lowest allelic error rate is obtained when 1,363 (50%) animals are in the reference and the rest in the test set. The mean allelic error rate ranges from 0.76% for the 35K SNP panel to 2.80% for evenly spaced 3K SNP panel using IMPUTE2. The mean imputation error rate for the cows using the bulls as reference ranges from 1.21 to 4.65% and for the bulls using the cows as reference ranges from 0.73 to 3.47% for different SNP densities using IMPUTE2 (Additional file 2).

#### Effect of relatedness between test and reference animals

The mean allelic error rates for the test animals with sire and without sire in the reference for all the 42 scenarios using IMPUTE2 are given in Additional file 2. In general test animals with sire in the reference have slightly lower allelic error rate of imputation (2.61% for with sire vs. 3.34% without sire averaged across all the scenarios). We further compared the error rate with kinship estimates of the test animals with the reference animals. The results for the 42 scenarios presented in Additional file 3 show that, in general, the mean allelic error rate decreases with the increase in the highest kinship of the test animals with the reference animals. This is more pronounced when the SNP panels in the test set are small and also when the reference size is small.

### Imputation between SNP chips

The mean allelic error rates of imputing SNP genotypes between different SNP chips obtained with IMPUTE2 are presented in Table 3. The results from BTA20 are given as an example. The mean allelic error rates of imputing 15K specific (205 SNPs) genotypes are 0.80%, 0.95% and 1.40% when 25%, 50% and 75% of the animals, respectively, are in the test set and the remainder of the animals with genotypes on 1529 SNPs (15K+50K) in the reference set. The mean allelic error rates of imputing 50K specific (1324 SNPs) genotypes are 2.85%, 3.15% and 4.25% when 25%, 50% and 75% of animals, respectively, are in the test set.

**Table 3:** Mean allelic error rate of imputing SNP genotypes between different SNP chips obtained with IMPUTE2

**Scenario**	**Animals masked (%)**	**N animals total**	**N animal reference**	**N animals test**	**N SNP**	**N**	**%**	**Mean allelic error rate (%)**
						**snp**	**snp**	
						**masked**	**masked**	
15K by 50K	25	1419	1065	354	1529	205	13	0.80
	50	1419	710	709	1529	205	13	0.95
	75	1419	355	1064	1529	205	13	1.40
50K by 15K	25	1419	1065	354	1529	1324	87	2.85
	50	1419	710	709	1529	1324	87	3.15
	75	1419	355	1064	1529	1324	87	4.25
25K by 50K	25	431	324	107	1652	328	20	1.50
	50	431	216	215	1652	328	20	1.85
	75	431	108	323	1652	328	20	2.75
50K by 25K	25	431	324	107	1652	1324	80	2.75
	50	431	216	215	1652	1324	80	2.75
	75	431	108	323	1652	1324	80	4.55

The results are shown for three SNP chips viz. 15K, 25K and 50K and chromosome 20.

Similarly the mean allelic error rates of imputing of 25K specific (328 SNPs) genotypes are 1.50%, 1.85% and 2.75% when 25%, 50% and 75% of the animals, respectively, are in the test set. The respective mean allelic error rates of imputing 50K specific (1324 SNPs) genotypes are 2.75%, 2.75% and 4.55%. The error rates in these scenarios are slightly higher compared to the above mentioned corresponding scenarios including 15K, possibly due to a lower number of animals in the reference and the test sets. Overall the results indicate that a reasonable accuracy of imputation for untyped SNP genotypes can be achieved when combining datasets genotyped with these SNP chips.

### Comparison of methods for imputation up to 800K

**Figure 3:**
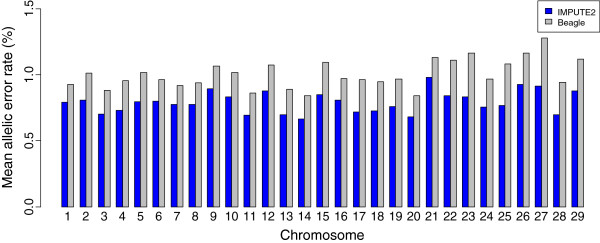
Mean allelic error rate (%) of imputing high density SNPs (800K) using 49K SNPs in the test set comparing two methods of imputation across all autosomes.

Only two methods (Beagle and IMPUTE2) were compared for imputing genotypes up to 800K using 50K. We did not include fastPhase in these comparisons because of the long computation time and the lower accuracy of fastPhase observed in the previous analyses within the 50K dataset. The chromosome-wise comparisons of the accuracies of the two methods are presented in Figure 3. The mean allelic error for imputing genotypes across different chromosomes ranges from 0.67% for BTA14 to 0.97% for BTA21 using IMPUTE2 and 0.84% for BTA14 to 1.28% for BTA27 when using Beagle. The mean error rates are slightly higher for smaller chromosomes (21–29) compared to larger chromosomes for both the methods (Figure 3). Genome-wide mean allelic error rate is less than 1% for both the methods (0.79% for IMPUTE2 and 0.99% for Beagle). Since IMPUTE2 outperformed Beagle for all the autosomes, this method was used for the analyses presented in the following sections.

### Comparison of 2-tiered and 3-tiered approaches for imputation up to 800K

**Figure 4:**
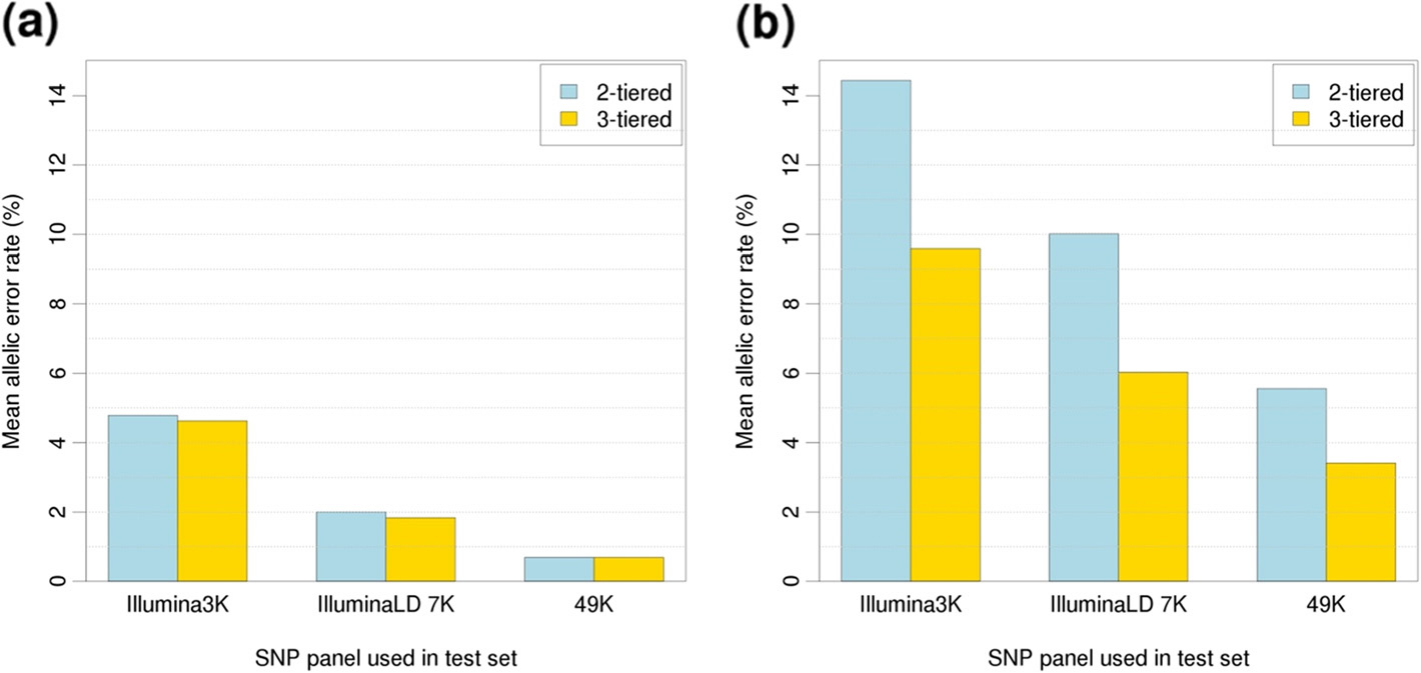
**Mean allelic error rate (%) of imputing high density SNPs (800K) using different number of SNPs in the test set by 2-tiered and 3-tiered approach.** Scenario (**a**) included 425 reference and 420 test animals, scenario (**b**) included 41 reference and 420 test animals. In the 3-tiered approach, an additional set of 2205 bulls with 50K data is included as middle tier in both scenarios (**a**) and (**b**). The results shown are for chromosome 20.

Accuracies of imputation using a 2-tiered and 3-tiered approach (Figure 1) to impute up to 800K SNP genotypes with IMPUTE2 are shown in Figure 4. The results presented are for BTA 20 as an example. Across all the scenarios examined, the mean allelic error rate of imputation is lower in the 3-tiered approach compared to the 2-tiered (Figure 4). The mean allelic error rate of imputing up to 800K decreases from 4.78% in the 2-tiered approach to 4.62% in the 3-tiered when Bovine3K SNP panel are used in the test animals (Figure 4a). A similar decrease in the mean allelic error rate is observed for BovineLD 7K panel (2.00% to 1.84% for 2-tierd and 3-tiered approaches, respectively). However, the relative improvement in allelic error rate from 2-tiered to 3-tiered are marginal for imputing up to 800K genotypes from 49K genotypes (0.689% to 0.688% for 2-tierd and 3 tiered approaches, respectively).

We further tested the accuracy of imputation using a smaller number of animals in the top tier. The mean allelic error rates for all scenarios are much higher when a small number of animals (41 animals, 5% of 825 cows) is included in the top tier (Figure 4b). The mean allelic error rates for the 2-tiered approach ranges from 5.55% using 49K to 14.43% for using the Bovine3K panel in the test set. However, there are larger decrease in the error rates of imputation using the Bovine3K (14.43% to 9.58%), BovineLD 7K (10.01% to 6.03%) and 49K (5.55% to 3.41%), by including a middle tier of 2205 bulls with 50K genotypes when the top reference tier is small.

To further test the potential of using 800K for imputing even higher density genotypes (e.g. up to 3 million or whole genome sequence) we tested accuracy of imputing every 10^th^ SNP and 100^th^ SNP by masking these SNP genotypes in 50% of the 825 cows genotyped with 800K using BTA20 as an example. The imputation accuracies for masked genotypes were 99.78% and 99.80% for every 10^th^ and 100^th^ SNP, respectively. However, such a large number of animals genotyped with very high-density SNP arrays or whole genome sequence may not be available in immediate future. We also tested a scenario when a smaller reference set (41 animals) was used and the accuracies of imputed genotypes were 98.00% and 98.44% for imputing every 10^th^ and 100^th^ SNP, respectively suggestive that ultra high-density and whole genome sequence may also be imputed with a very high level of accuracy from a commercial high-density SNP array.

### Accuracy of DGV prediction based on actual and imputed genotypes using 50K dataset

Accuracy of DGV prediction of five dairy traits using actual 50K, Bovine3K and BovineLD 7K genotypes are compared with DGV predictions using imputed genotypes up to 50K in Table 4. Accuracy of DGV predictions based on imputed genotypes are very close (within 2.4%) to those obtained using the actual 50K genotypes when all the training set bulls are used in the reference set for imputation (scenario A Table 4). Accuracies of DGV using imputed genotypes are slightly lower when smaller reference set is used for imputation (scenario B Table 4). In scenario B all the test bulls and most of the training bulls have imputed genotypes. The lower accuracies under scenario B are more evident for Bovine3K which has much higher mean allelic error rate (5.52%). In all the scenarios the accuracies of DGV from imputed genotypes are higher than from the actual smaller subset of SNPs on which the imputation is based. These results indicate that imputed genotypes for both training and test set can be used without any loss of accuracies of DGV prediction especially when BovineLD 7K is used.

**Table 4:** Accuracy of prediction of direct genomic value (DGV) for 5 dairy traits based on Bovine3K, BovineLD 7K, 50K, imputed up to 50K, imputed up to 800K and imputed 800K-dosage

**Genotypes used**	**Mean allelic error rate (%) of imputation**	**Milk volume**	**Fat yield**	**Protein yield**	**Survival**	**Daughter fertility**
50K	-	0.540	0.527	0.499	0.224	0.251
Subset Bovine3K	-	0.444	0.464	0.429	0.187	0.200
Subset Bovine LD 7K	-	0.481	0.516	0.443	0.186	0.232
50K-imputed (Test imputedA^A^ using Bovine3K)	3.86	0.533	0.523	0.496	0.200	0.244
50K-imputed (Test imputed^A^ with BovineLD)	2.30	0.546	0.531	0.507	0.214	0.246
50K-imputed (Train & Test imputed^B^ using Bovine3K)	5.52	0.505	0.515	0.481	0.207	0.245
50K-imputed (Train & Test imputed^B^ using BovineLD)	3.06	0.530	0.524	0.492	0.209	0.248
800K-imputed^C^	-	0.558	0.530	0.526	0.232	0.256
800K-dosage^C^	-	0.554	0.525	0.520	0.229	0.253

^A^Genotypes of 452 young bulls with subset of original SNPs were imputed (using IMPUTE2) up to 50K using 1753 bulls as reference set. Hence for DGV prediction entire test set (452 young bulls) had imputed genotypes and all the training bulls (1753) had actual 50K genotypes.

^B^Genotypes of 2055 bulls with subset of original SNPs were imputed (using IMPUTE2) up to 50K using 136 bulls as reference set. Hence for DGV prediction the entire test set (452 young bulls) and 1617 bulls out of the training set of 1753 bulls had imputed genotypes.

^C^Data on 2205 bulls genotyped for 50K were imputed using IMPUTE2 up to 800K using 845 cows genotyped on 800K as reference.

### Accuracy of DGV prediction based on 800K imputed data

Table 4 further presents the results on accuracies of DGV prediction using imputed genotypes up to 800K. The accuracies of DGV prediction using the most likely genotype (800K-imputed) and allele dosage (800K-dosage) are quite similar viz. 0.558 and 0.554 for milk yield, 0.530 and 0.525 for protein yield, 0.526 and 0.520 for fat yield, 0.232 and 0.229 for survival and 0.256 to 0.253 for daughter fertility, respectively. Overall there is only a small improvement in DGV prediction using the imputed 800K genotypes over the actual 50K genotypes.

## Discussion

With the rapid development of higher density SNP chips for cattle, it is now common to have population samples genotyped with different SNP chips. We have presented different strategies for utilising such heterogenous SNP datasets efficiently. We compared accuracies of imputation within and across SNP chips and the accuracy of genomic prediction using imputed genotypes.

IMPUTE2 gave higher accuracies of imputation compared to Beagle and fastPhase. fastPhase may provide comparable accuracy when the reference panel is small and the SNP densities used in the test set is high. However fastPhase required more computing time compared to Beagle and IMPUTE2. For example for scenario 1 (Additional File 2), using a Linux machine with AMD Opteron Processor 6136, IMPUTE2, Beagle and fastPhase took 2.36, 6.19 and 20.7 hours of computing time and used 100MB, 807MB, 112MB RAM, respectively. Computation time on a multiprocessor machine can be reduced by dividing the chromosome into smaller segments. However, using IMPUTE2, we observed that accuracy was slightly higher when the whole chromosome was imputed in a single run (not shown). This may possibly be due to the extended linkage disequilibrium present in the bovine genome [42] which allows for better definition of long-range haplotypes when the whole chromosome is used.

Our estimates of mean allelic error of imputing up to 50K from evenly spaced 3K panel (2.8%) were lower compared to Bovine3K (3.3%) which may be because of the higher number of SNPs with higher MAF in evenly spaced 3K SNP panel. These estimates are comparable to the range of 2.1 to 5.5% reported by Dassonneville et al. [32] for Bovine3K and 3 to 4% obtained by Zhang et al. [30] for evenly spaced 3000 SNPs using DAGPHASE. We found an increase in the accuracy of imputation with an increase in the number of animals in the reference set. However, we tested only up to 1,363 animals in the reference. Larger reference sets might further improve accuracy of imputation.

We showed that 800K genotypes could be imputed with low allelic error using 50K genotypes (0.79% for all autosomes). Most of the SNPs had low error rate. However, we noted a very small proportion of the SNPs with higher imputation error than expected. For example we found 12 SNPs on BTA20 which had an allelic error rate of larger than 5%. We suspect that these SNPs may have incorrect positions on UMD3.1 assembly or contain errors in genotyping call itself. The mean error rates reported throughout this study include all such SNPs. If wrong map assignment and genotypic error of SNPs have a significant effect on the accuracy of imputation process is not known, but should be considered in future studies.

We showed that using additional reference panel genotyped with medium-density SNP chip in a 3-tiered framework increased the accuracy of imputation especially when the main reference panel was small. The additional gain in the accuracy of imputation in the 3-tiered approach may be due to better definition of haplotypes with the availability of large number of samples in the combined reference [38]. Our results suggest that increasing the size of the reference panel by including animals genotyped with different SNP chips in a tiered framework can improve the accuracy of imputation. We used population based methods for imputation and showed that these used relationship information indirectly. The degree of kinship between animals in test and reference set has a significant effect on the accuracy of imputation and as such can be strategically optimised in selecting animals to be genotyped if pedigree information is available. A number of other programs have been used for imputation ( [43-45], [33]) which use pedigree information directly along with haplotype data and these can be more efficient when required family information is available. Johnston et al. [44] suggested a blending approach that combined the strength of various programs available. Development of multi-tiered imputation strategies that utilises pedigree information seems promising when the animals genotyped with heterogenous SNP panels and up to whole genome sequences are available.

Using imputed genotypes up to 50K increased the accuracy of genomic selection compared to just using the smaller SNP subsets used for imputation. Similar observations were made by Johnston et al. [44] and Weigel et al. [46]. Therefore, using genotype imputation would increase return on investment when a larger proportion of the population is genotyped with lower density SNP panels.

By testing the utility of imputed 800K genotypes *i.e.* best guess genotypes and dosages of the B-allele, we showed that the accuracy of genomic prediction from imputed 800K genotypes was only marginally better compared to using 50K genotypes. Although we cannot compare these accuracies with the actual 800K genotypes in this study, however, mean allelic error rate of imputation up to 800K using 50K in the test samples was very small (0.79%). These error rates were obtained by using 425 cows in the reference set. The results of imputing up to 50K (Figure 2) show that using larger reference can improve accuracy of imputation even further. Moreover additional analyses within the 50K dataset indicate that small error rates of the imputed genotypes will have no notable effect on the accuracy of genomic selection. Hence we believe that presented accuracies of genomic prediction with imputed 800K genotypes are comparable to the actual 800K genotypes. However, we have only used one method for genomic prediction and it is possible that other methods may utilise higher density genotype more efficiently (e.g. [31], [47]). High-density SNP genotypes are likely to be useful for genome-wide association studies and across study meta-analysis of SNP-trait relationships. Further studies are required to see the utility of imputed genotypes to discover and map the casual mutation affecting phenotypes in dairy cattle.

## Conclusions

IMPUTE2 had the highest accuracy of the three imputation methods examined. Accuracy of imputation increases with the number of SNPs in the test set, increase in the number of samples in the reference set and presence of closely related animals in the reference. 800K SNP genotypes can be imputed with very high accuracies from 50K SNP genotypes and with slightly lower accuracies from lower density SNP panels (e.g. 3K, 7K). The accuracy of imputation is improved using a 3-tiered approach, which used an additional middle tier of 50K, compared to 2-tiered approach, especially when the top panel of animals genotyped with 800K SNPs is small. There is no appreciable loss in accuracy of genomic prediction using imputed 50K SNP genotypes derived from the commercial 3K or 7K panels compared to using the actual 50K SNP genotypes and both perform substantially higher than using 3K or 7K genotypes. Our results show that imputation from lower density SNP panels is a cost effective strategy for genomic selection. There is only a small gain in the accuracy of genomic prediction when using imputed 800K genotypes compared to actual 50K genotypes.

## Abbreviations

(GS): Genomic selection; (DGV): Direct genomic values; (SNP): Single nucleotide polymorphism; (LD): Linkage disequilibrium; (HMM): Hidden Markov Model; (HWE): Hardy-Weinberg Equilibrium; (MAF): Minor allele frequency; (DTD): Daughter trait deviations.

## Competing interests

The authors declare that they have no competing interests.

## Authors' contributions

MSK conceived study, contributed in its design, data collection, analyses and was the primary author for assembling the manuscript. GM contributed in the analysis and preparation of the manuscript. BJH contributed in the design, data acquisition, QC and preparation of the manuscript. HWR contributed in project concept, design, interpretation and manuscript preparation. All authors read and approved the final manuscript.

## Supplementary Material

Additional file 1 **Figure S1.** Distribution of pedigree kinship among animals within different datasets shown as boxplots. Click here for file

Additional file 2 **Accuracy of imputation of genotypes (%) and mean allelic error rate (%) up to 50K using three imputation methods. **This file presents the results from different scenarios of imputation up to 50K. These scenarios were generated by using different proportion of animals in reference and test sets for varying SNP density (3K, 5K, 10K, 20K and 35K evenly spaced and Illumina Bovine3K) in the test set. The scenarios 1–6 used 1363 (50%) bulls, scenarios 7–12 used 681 (25%) bulls, scenarios 13–18 used 272 (10%) bulls, scenarios 19–24 used 136 (5%) bulls and scenarios 25–30 used 27 (1%) bulls in the reference set and the rest of animals in the test set. The scenarios 31–36 used all the bulls in the reference and all the cows in the test set. The scenarios 37–42 used all the cows in the reference and all the bulls in the test set. The results shown are for chromosome 20. Click here for file

Additional file 3 **Effect of pedigree kinship between test and reference animals on the mean allelic error rate (%) of imputation.** This file presents the results of association of kinship with error rate of imputation in the form of bar charts from 42 scenarios of imputation up to 50K as given in Additional file 2. On Y-axis, is highest kinship estimate of a test animal with any of the reference animals and is presented as four interval categories viz. 0.0-0.01, 0.01-0.1, 0.1-0.2 and 0.2-0.4. On X-axis is the mean allelic error rate (%) on imputation. Click here for file
