# Supplementary material for: Strategies and utility of imputed SNP genotypes for genomic analysis in dairy cattle
Source: BMC Genomics. 2012 Oct 8;13:538. doi: 10.1186/1471-2164-13-538 (PMC3531262; doi:10.1186/1471-2164-13-538)
Supplement: Additional file 1 — Figure S1. Distribution of pedigree kinship among animals within different datasets shown as boxplots. [file 1471-2164-13-538-S1.doc]

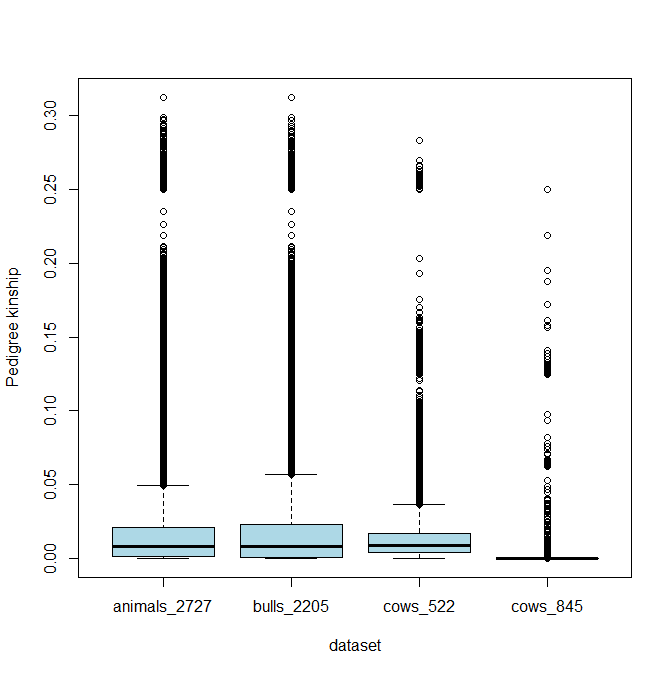


Figure S1. Distribution of pedigree kinship among animals within different datasets shown as boxplots.
