# Supplementary material for: Strategies and utility of imputed SNP genotypes for genomic analysis in dairy cattle
Source: BMC Genomics. 2012 Oct 8;13:538. doi: 10.1186/1471-2164-13-538 (PMC3531262; doi:10.1186/1471-2164-13-538)
Supplement: Additional file 3 — Effect of pedigree kinship between test and reference animals on the mean allelic error rate (%) of imputation. This file presents the results of association of kinship with error rate of imputation in the form of bar charts from 42 scenarios of imputation up to 50K as given in Additional file 2. On Y-axis, is highest kinship estimate of a test animal with any of the reference animals and is presented as four interval categories viz. 0.0-0.01, 0.01-0.1, 0.1-0.2 and 0.2-0.4. On X-axis is the mean allelic error rate (%) on imputation. [file 1471-2164-13-538-S3.pdf]

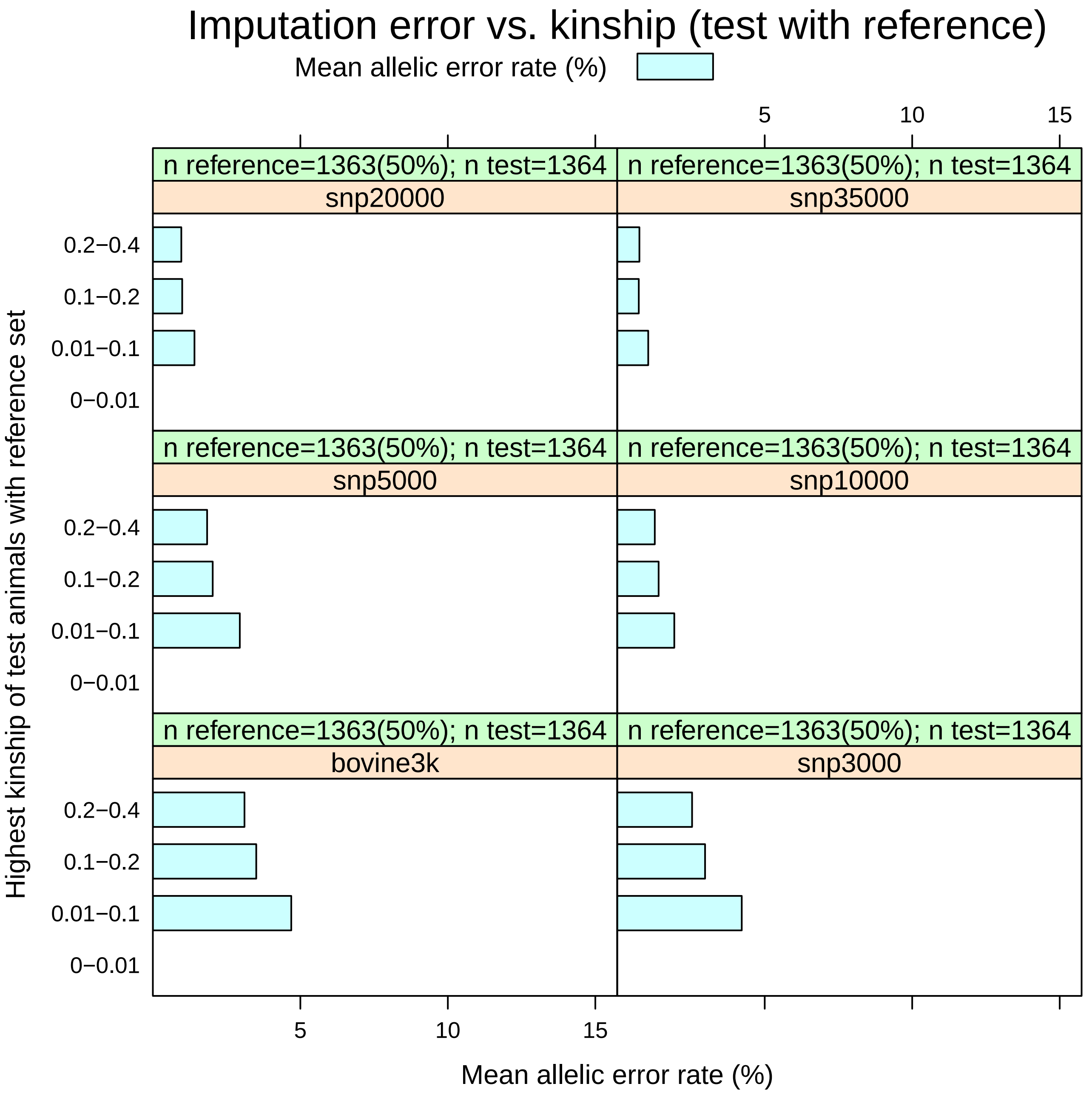

Imputation error vs. kinship (test with reference)
Mean allelic error rate (%)
n reference=1363(50%); n test=1364
snp20000
snp35000
snp5000
snp10000
bovine3k
snp3000
0.2−0.4
0.1−0.2
0.01−0.1
0−0.01
5
10
15
Highest kinship of test animals with reference set
Mean allelic error rate (%)

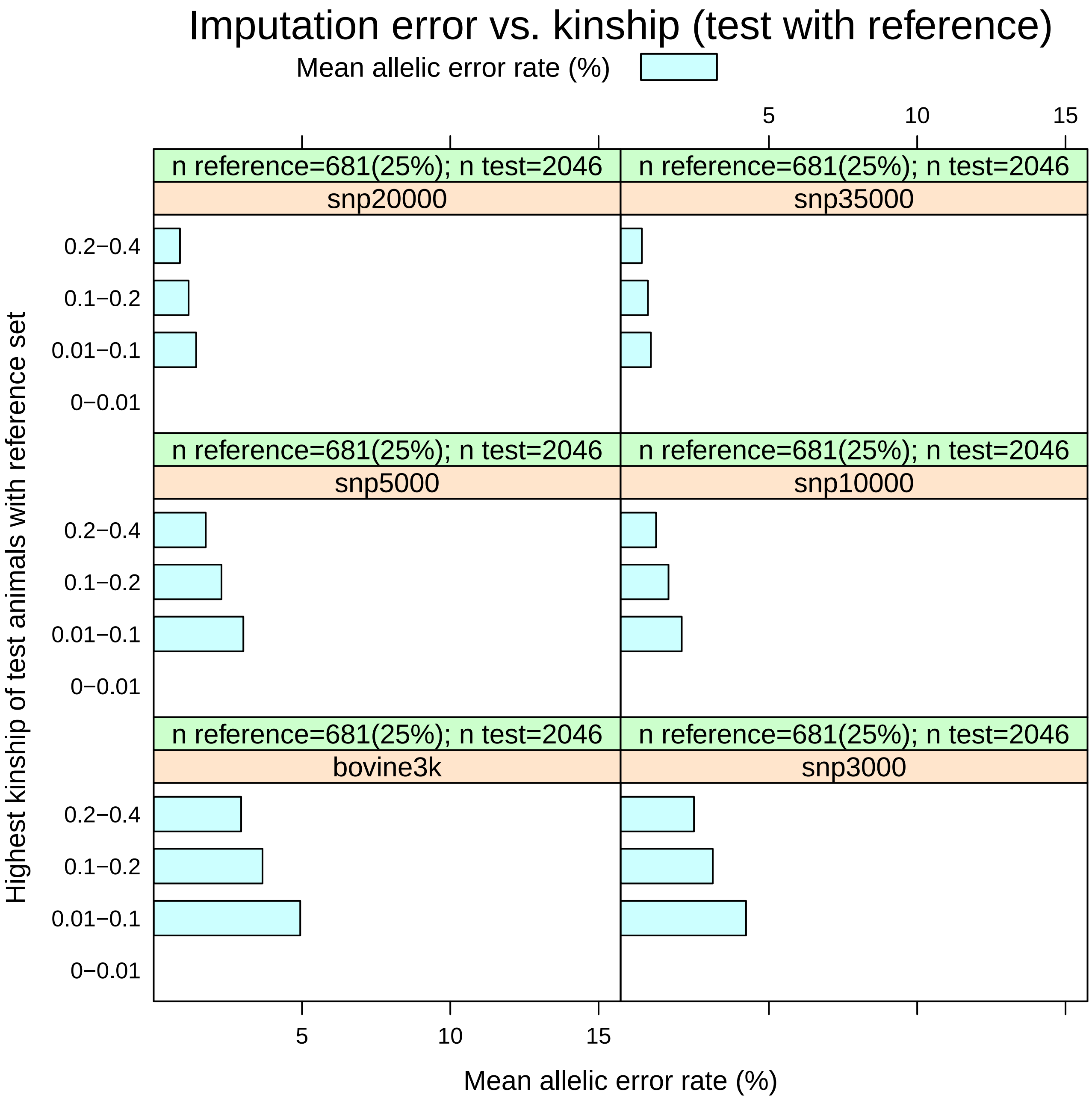
Imputation error vs. kinship (test with reference)
Mean allelic error rate (%)
n reference=681(25%); n test=2046
snp20000
snp35000
snp5000
snp10000
bovine3k
snp3000
0.2−0.4
0.1−0.2
0.01−0.1
0−0.01
5
10
15
Highest kinship of test animals with reference set
Mean allelic error rate (%)

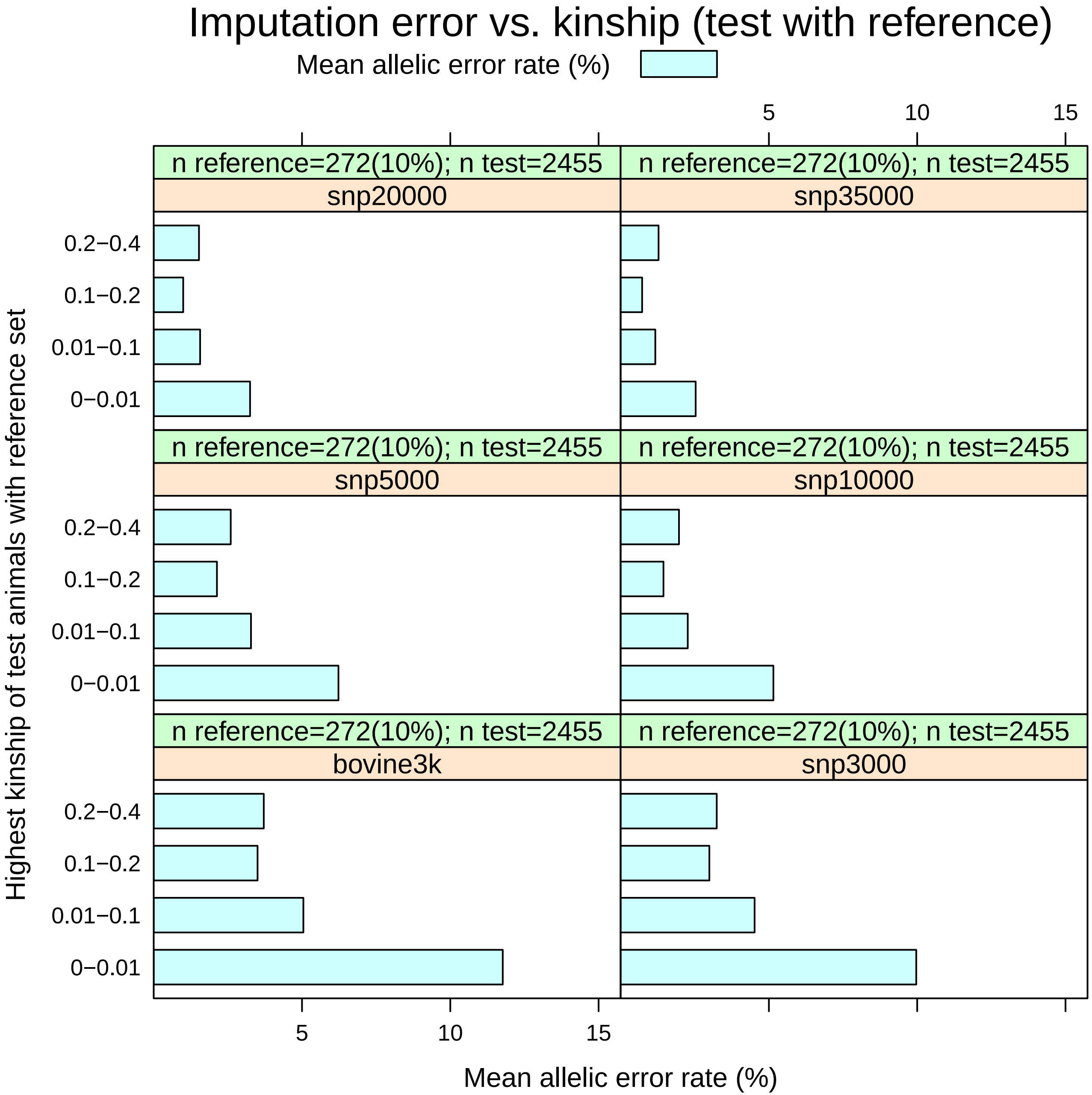
Imputation error vs. kinship (test with reference)
Mean allelic error rate (%)
n reference=272(10%); n test=2455
snp20000
snp35000
snp5000
snp10000
bovine3k
snp3000
0.2−0.4
0.1−0.2
0.01−0.1
0−0.01
5
10
15
Highest kinship of test animals with reference set
Mean allelic error rate (%)

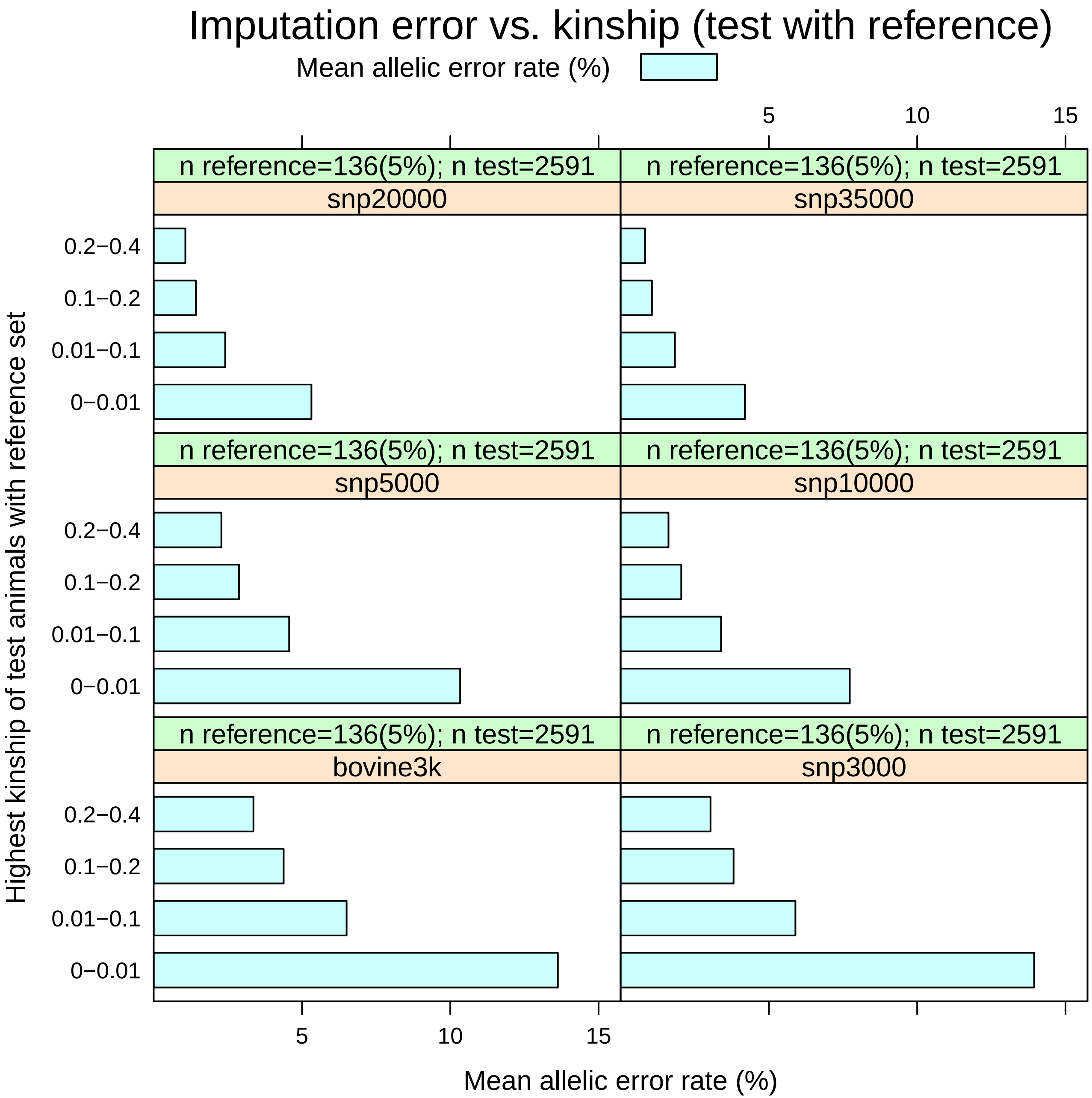

Imputation error vs. kinship (test with reference)
Mean allelic error rate (%)
n reference=136(5%); n test=2591
snp20000
snp35000
snp5000
snp10000
bovine3k
snp3000
0.2−0.4
0.1−0.2
0.01−0.1
0−0.01
5
10
15
Highest kinship of test animals with reference set
Mean allelic error rate (%)

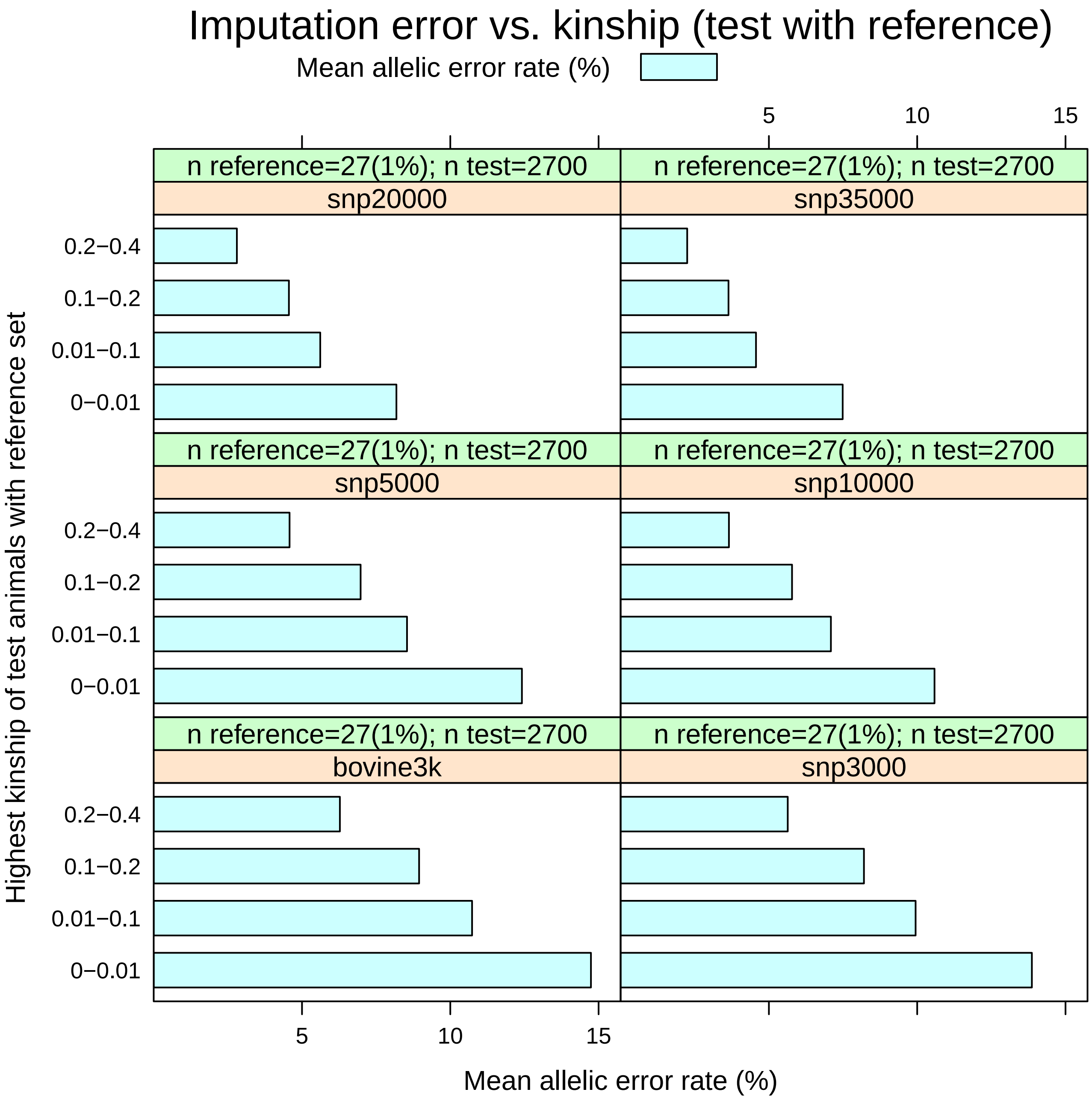

Imputation error vs. kinship (test with reference)
Mean allelic error rate (%)
n reference=27(1%); n test=2700
snp20000
snp35000
snp5000
snp10000
bovine3k
snp3000
0.2−0.4
0.1−0.2
0.01−0.1
0−0.01
5
10
15
Highest kinship of test animals with reference set
Mean allelic error rate (%)

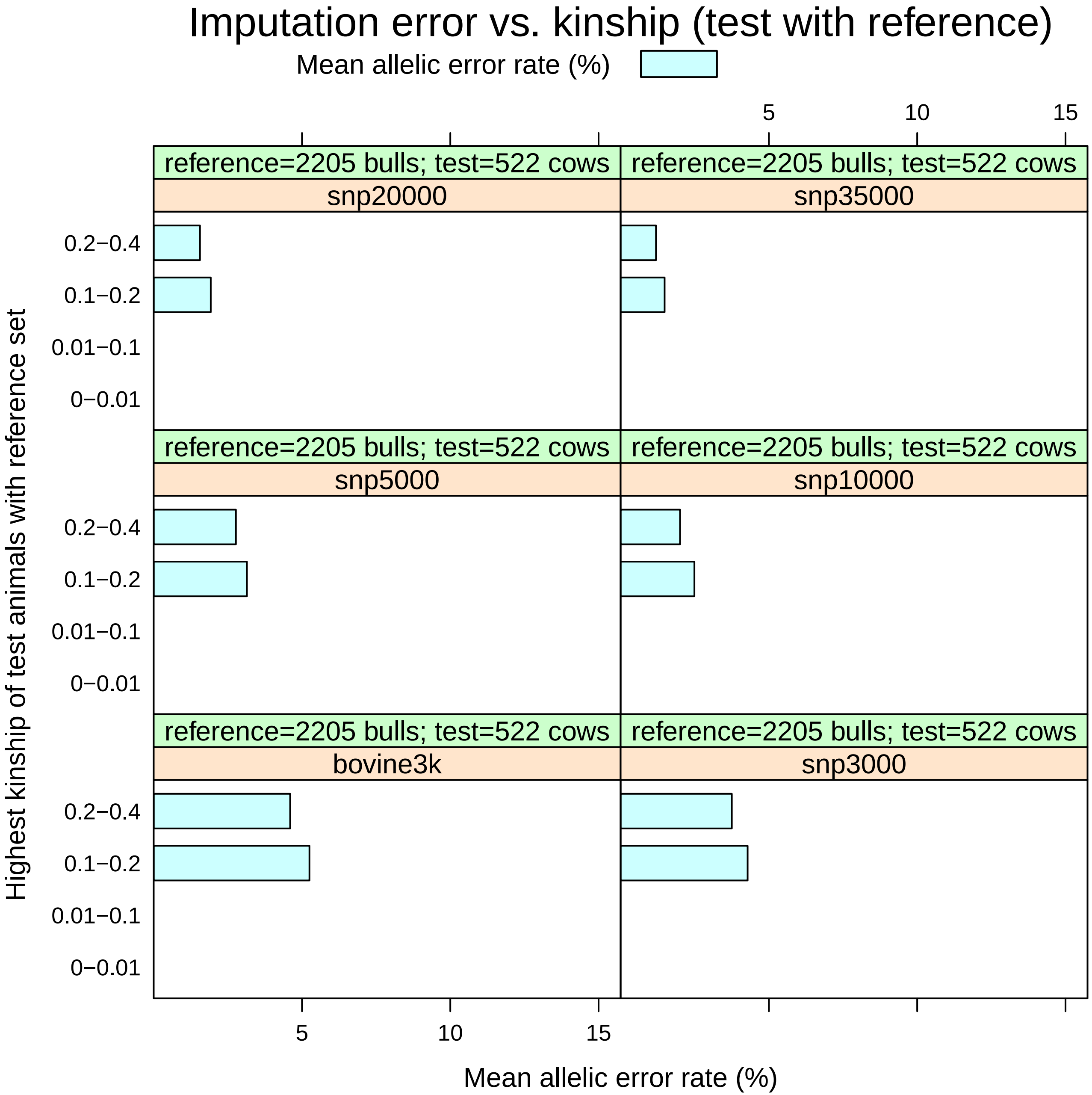

Imputation error vs. kinship (test with reference)
Mean allelic error rate (%)
reference=2205 bulls; test=522 cows
snp20000
snp35000
snp5000
snp10000
bovine3k
snp3000
0.2−0.4
0.1−0.2
0.01−0.1
0−0.01
5
10
15
Highest kinship of test animals with reference set
Mean allelic error rate (%)

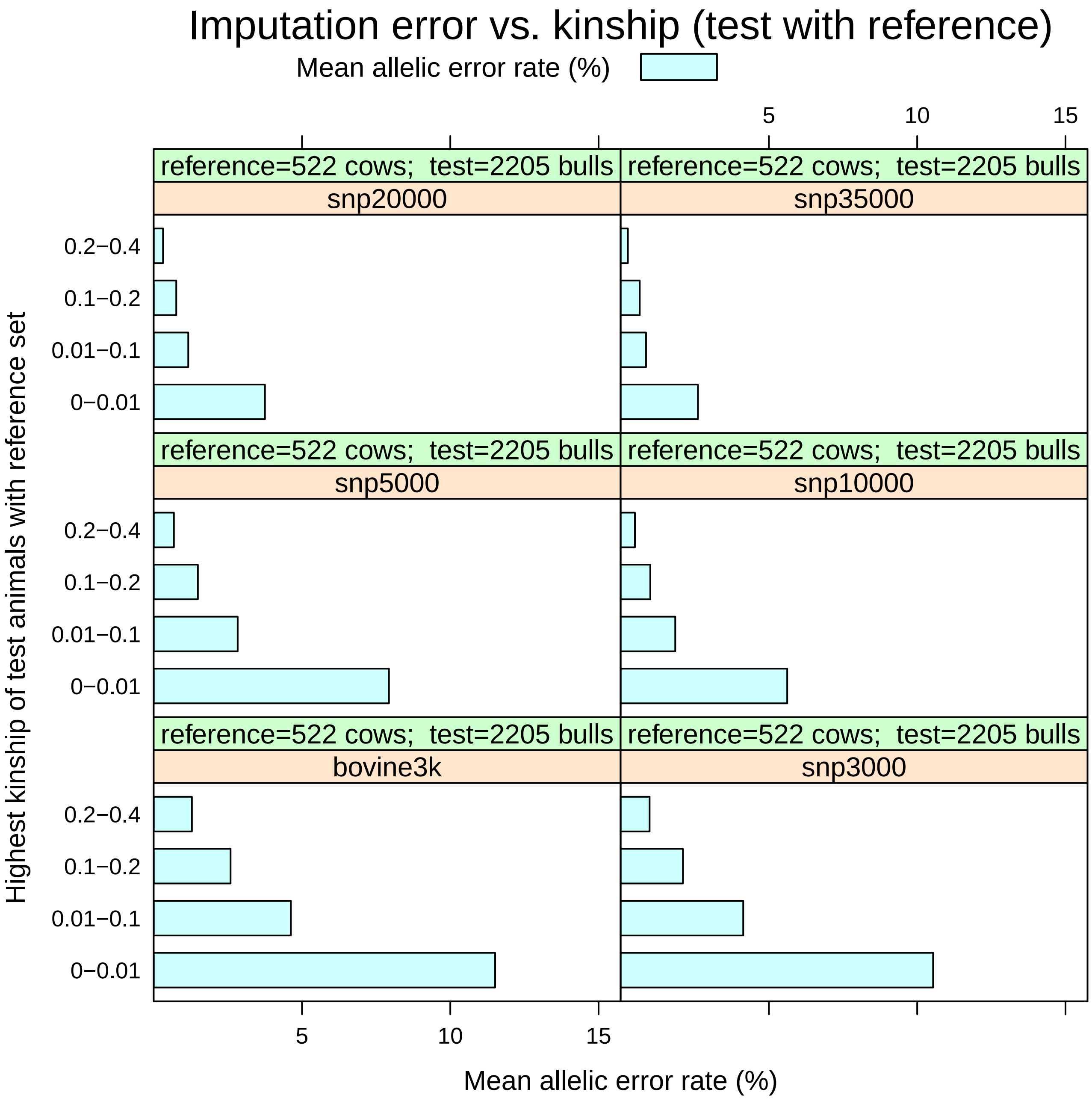

Imputation error vs. kinship (test with reference)
Mean allelic error rate (%)
reference=522 cows; test=2205 bulls
snp20000
snp35000
snp5000
snp10000
bovine3k
snp3000
0.2−0.4
0.1−0.2
0.01−0.1
0−0.01
5
10
15
Highest kinship of test animals with reference set
Mean allelic error rate (%)
